# Clinical and Optical Coherence Tomography Evidence of Aqueous Humor Flow from the Suprachoroidal Space to Conjunctival Lymphatics

**DOI:** 10.3390/vision7030059

**Published:** 2023-09-05

**Authors:** Vinod Kumar, Andrey Igorevich Bezzabotnov, Zarina Shaykuliyevna Rustamova, Galina Nikolaevna Dushina, Kamal Abdulmuhsen Abu Zaalan, Ahmad Saleh Soliman Shradqa, Mikhail Aleksandrovich Frolov

**Affiliations:** 1 Department of Eye Diseases, Medical Institute, Peoples’ Friendship University of Russia Named after Patrice Lumumba, 6 Mikluho-Maklaya St., 117198 Moscow, Russia; shaykuliyevna0294@gmail.com (Z.S.R.); dushina_galina@mail.ru (G.N.D.); frolovma@rambler.ru (M.A.F.); 2 Centre of Eye Microsurgery “PRO Zrenie”, 1 Gorshina Str., 141407 Khimki, Russia; magnawer@yandex.ru (A.I.B.); 79686112302@mail.ru (K.A.A.Z.); sh1988moscow@gmail.com (A.S.S.S.)

**Keywords:** glaucoma, open-angle glaucoma, collagen implant, glaucoma surgery, bleb less glaucoma surgery, bleb-independent glaucoma surgery, suprachoroidal space, uveolymphatic aqueous humor outflow, conjunctival lymphatic vessels, conjunctival lymphatics

## Abstract

A surgical technique was developed to enhance aqueous humor (AH) flow through the non-trabecular outflow pathway by rerouting it from the anterior chamber (AC) to the suprachoroidal space (SCS) without detaching the ciliary body from the scleral spur. Medium- and long-term surgical outcomes were retrospectively analyzed in a case series of 58 glaucoma patients. At 6, 12, and 24 months, the mean IOP decreased from 27.8 ± 8.3 to 14.9 ± 5.0 mmHg, median 15.0 (25th percentile (p^25^)13.0; 75th percentile (p^75^) 18.0) and 15.2 ± 3.3 mmHg, and hypotensive medication use reduced from a median (p^25^; p^75^) of 3 (2; 3) to 0 (0; 2), 0 (0; 2), and 0 (0; 1.5), respectively. Intra- and postoperative complications were few and manageable. Following surgery, no bleb formation occurred in any of the cases (as confirmed by optical coherence tomography). Conjunctival lymphatic vessels (CLVs) developed in 50% of eyes (29/58). Clinically, they developed directly from sclera and had no connection to the surgical site. Analysis further showed that the development of CLVs and their longer visibility period had poor prognostic value for IOP control. If the fluid flow from the SCS to CLVs was resistance-free, no CLV development was evident. However, if any resistance existed in the flow, the fluid accumulated in lymphatics, resulting in their engorgement. The proposed technique was safe and effective in decreasing IOP in glaucoma patients by enhancing AH flow from the SCS to CLVs via connecting intrascleral microchannels.

## 1. Introduction

Glaucoma is a neurodegenerative ocular disease. The imbalance between aqueous humor (AH) production and outflow causes elevation of intraocular pressure (IOP). AH flows out from the anterior chamber (AC) via trabecular (traditional) and non-trabecular (non-traditional) pathways [1].

Penetrating and non-penetrating filtering glaucoma surgeries performed for the management of open-angle glaucoma (OAG) aim to create an artificial pathway for AH outflow. Penetrating glaucoma surgeries are effective for a long-term decrease in IOP but may produce various complications [2,3]. Non-penetrating glaucoma surgeries are safer; however, they produce a short-term hypotensive effect, and Nd:YAG laser goniopuncture is a mandatory step to maintain the hypotensive effect [4,5]. Both classes of glaucoma surgeries are bleb-dependent. The AH, after leaving the AC, accumulates in a subconjunctival space (bleb), from where it may percolate into the cut ends of Schlemm’s canal, outflow to the suprachoroidal space (SCS), or become absorbed into the conjunctival blood vessels or lymphatics [6,7]. Alternatively, if the bleb is thin-walled, it passes directly across the conjunctiva into the tear layer [8,9]. Fibrosis occurring at the operation site is the main cause for bleb failure. Recently, antimetabolites have been used both intra- and postoperatively to prevent the occurrence of fibrosis; however, their use has increased the risk of the development of certain complications, such as bleb leakage, blebitis, and endophthalmitis [10].

The non-trabecular outflow pathway has good potential to decrease IOP. Various surgical techniques and modifications of the existing techniques in the field have been proposed in the literature, and different devices have been used in clinical practice, to date, to enhance AH outflow via this pathway. However, the surgical outcomes of these techniques and devices are unpredictable [11,12,13,14,15,16]. In most of these techniques, performing surgical cyclodialysis is mandatory.

We developed a surgical technique aimed at rerouting AH outflow from the AC to the SCS without detaching the ciliary body from the scleral spur to decrease IOP in glaucoma patients and, in a pilot study, we reported the short-term surgical outcomes, demonstrating a decrease in IOP without the formation of a bleb and the development of conjunctival lymphatic vessels (CLVs) in 40% of the examined cases [17,18]. In this paper, we report the medium- and long-term surgical outcomes of the technique that we employed, and we provide clinical and optical coherence tomography (OCT) evidence for AH outflow from the SCS to CLVs.

## 2. Materials and Methods

In this retrospective, non-comparative, and non-control interventional case series, medical records of 58 patients having undergone glaucoma surgery were analyzed. Glaucoma surgery was performed as a standalone procedure in 18 eyes (31%), and in combination with cataract surgery in 40 eyes (69%). One surgeon (V.K.) performed all of the operations between 1 January 2020 and 31 December 2021. 

The inclusion criteria were OAG, decompensated IOP following previous glaucoma surgeries, medically uncontrolled IOP, OAG in pseudophakic eyes, and a minimum postoperative follow-up period of 25 weeks.

The exclusion criteria were angle-closure glaucoma, secondary glaucoma, intraocular lens (IOL) dislocation with decompensated IOP, patients on anticoagulants, and patients with micro-perforations of the trabecular meshwork (TM) that occurred during surgery. 

Prior to surgery, all patients underwent a comprehensive ophthalmological examination. Visual acuity (VA) was assessed using Snellen’s chart. The VA values were converted to a logarithm of the minimum angle of resolution (logMAR). IOP was measured using an iCare tonometer (ic100, Finland Oy, Vantaa, Finland) [19]. The median values of three consecutive measurements were considered [20]. The IOP values were adjusted for corneal thickness using the “cornea analysis” application of spectral domain optical coherence tomography (OCT) (SOCT Copernicus Revo 80, OPTOPOL Technology Sp.z.o.o., Zawiercie, Poland). The patients’ field of vision was tested on a perimeter Perigraph Perikom (Spetsmedpribor Co. Ltd., Moscow, Russia). An OCT glaucoma analysis was conducted, provided that the native lens condition permitted this.

Preoperatively, the patient’s ocular hypotensive medications were not washed out. Antibacterial (sol. levofloxacin 0.5%, 1–2 drops, 3 times per day) and anti-inflammatory (sol. bromfenac 0.09% once per day) medications were prescribed to patients for a period of three days prior to surgery.

### 2.1. Surgical Technique

The details of the surgical technique that we employed are described elsewhere [18]. The aim of the technique—to reroute AH outflow from the AC to the SCS without detaching the ciliary body from the scleral spur—was achieved, first by creating an intrascleral reservoir (ISR) to receive and accumulate AH from the AC. From the ISR, a suprachoroidal tunnel was created and a collagen implant (CI) was inserted into it, leaving one end in the ISR. The CI acted as a spacer for the ISR and suprachoroidal tunnel, and as a conduit for AH flow from the ISR to the SCS. Schlemm’s canal was deroofed without creating a window in the trabeculo-Descemet’s membrane, and a part of the juxtacanalicular connective tissue (JCT) was removed. Postoperatively, if the IOP was observed to be elevated, Nd:YAG laser trabeculotomy was performed at the surgery site to allow for resistance-free AH flow from the AC to the ISR and SCS. This was performed no earlier than postoperative days 7–10, allowing enough time for the conjunctiva to heal, thus preventing AH flow from the ISR to the subconjunctival space. Sclerectomy was performed in the posterior section of the ISR to expose more uveal tissue for the resorption of AH. 

The ophthalmic CI (MakMedi, Moscow, Russia) used in this case series was composed of collagen material obtained from porcine sclera. These biologically inert implants are hydrophilic. When immersed in fluid, they swell and thicken. The frontal dimensions remain unchanged. With time, the implants are slowly resorbed by tissue fluids. The implants are permitted for use in human beings. For the study procedure, implants measuring 0.1 × 2 × 5 mm were selected.

All surgeries were performed on an ambulatory basis. Following the surgery, a monocular eye pad was applied on the operated eye. Postoperatively, patients followed a standard protocol. Five hours after the surgery, the patients removed their eye pads and began the eye drop instillation process: dexamethasone (0.1% solution) eye drops were used three times per day for the first week, which was then reduced to one drop per week, and the instillation of levofloxacin eye drops (0.5% solution) was continued three times per day for one additional week. In the case of suture irritation, the patients were advised to apply an antibiotic ointment (ofloxacin ointment 0.3%) until the sutures were removed. The patients continued with the instillation of hypotensive medications. All patients attended the outpatient department on the day after surgery. In cases where a patient’s IOP was higher than the target IOP, they were advised to continue with the instillation of hypotensive medications, and if the IOP was lower than the target IOP, the patients were advised to terminate the instillation of eye drops. Conjunctival sutures were removed 7–10 days following surgery. On this day, if the IOP remained elevated, Nd:YAG laser trabeculotomy was performed, and the patient was instructed to stop instillation of anti-glaucoma medications. All patients were evaluated on days 1 and 7, and then at 1, 3, 6, 9, 12, 18, and 24 months. If laser trabeculotomy was performed, the first postoperative day was the day following the Nd:YAG laser trabeculotomy; otherwise, the first day was the day after the surgery. The postoperative assessment included VA assessment, tonometry, biomicroscopy, direct ophthalmoscopy, and gonioscopy. Wherever possible, the results were documented using a digital photo slit lamp. In each follow-up session, the surgical site and adjunct areas were evaluated using OCT.

A slit-lamp-mounted Nd:YAG laser (OptoYAG, Optotek Medical, Slovenia) and a single-mirror laser gonio lens (OLSLTF, Latina SLT Gonio Laser Lens, Ocular instruments, Bellevue, WA, USA) were used to perform Nd:YAG laser trabeculotomy. Usually, 3 or 5 millijoules of energy was sufficient to create one or more openings in the trabecular meshwork (TM). In a successful trabeculotomy case, the pulsatile movement of AH passing through it was detected.

**Figure 1:**
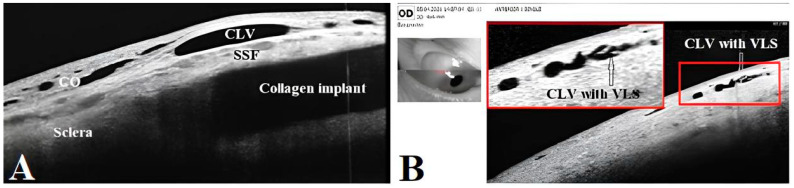
OCT evaluation: (**A**) OCT scan of the surgery site, showing the collagen implant in the intrascleral reservoir, covered by a superficial scleral flap (SSF) and conjunctiva (CO). No filtration bleb cavity can be observed over the implant or in the nearby area. A conjunctival lymphatic vessel (CLV) can be observed running horizontally over the surgical site. (**B**) OCT scan of conjunctiva adjacent to the surgical site showing a CLV with valve-like structures (VLSs) in its lumen (white and black arrows with black and white borders). CO = conjunctiva; CLV = conjunctival lymphatic vessel; SSF = superficial scleral flap; VLS = valve-like structure.

OCT images taken prior to and following the surgery were acquired by using the commercially available SOCT Copernicus Revo 80, using the technique described earlier [18], and were evaluated as described by Kawana K. et al. [21]. In Figure 1, an example of the OCT evaluation of the surgery and adjunct sites is presented.

The outcome measures were IOP change, the use of hypotensive medication(s), complications, and the need for a second surgery.

Eyes with mild OAG had a target IOP of at least 20% reduction from the baseline or IOP ≤ 21 mmHg (whichever was lower); eyes with moderate and severe OAG had a target IOP of at least 30% reduction from the baseline or ≤18 mmHg (whichever was lower) [22,23]. 

Complete success was assigned to a case if, at the last follow-up visit, the target IOP was achieved without the use of additional glaucoma medications. In the case of a qualified success, the target IOP was achieved with or without the use of glaucoma medications. Cases with partial success were those that achieved target IOPs with the use of additional hypotensive medications. A case was judged as a failure case if the patient’s IOP level was measured above the upper limit or below the lower limit during two consecutive visits, and if further glaucoma interventions were required. In cases with preoperative medically controlled IOP, the IOP reduction was assessed by the percentage reduction in the IOP value. The IOP reduction had to be greater than 20% for it to be considered a success. Fixed-combination glaucoma medications were counted as two separate medications.

### 2.2. Statistics

The Shapiro–Wilk test was used to determine the distribution of continuous variables. Categorical variables were described as the frequency with percentages. Continuous and discrete variables were described as means with standard deviations (SDs) or medians with percentiles (p^25^; p^75^). A paired Student’s *t*-test was applied to calculate the significance between two paired groups for parametric variables, and a Wilcoxon rank sum test was used for non-parametric variables. VA and medications were examined using Friedman and Wilcoxon signed-rank tests. The success of the treatment was expressed by the Kaplan–Meier curve, and *p*-values below 0.05 were considered statistically significant. SPSS Statistics (IBM SPSS Statistics, Armonk, NY, USA) 28.0.0.0 software for Windows 7 and the Excel application of Microsoft Office 365 (Microsoft Corporation, Redmond, WA, USA) were used for statistical processing purposes.

## 3. Results

The demographic and clinical data of 58 patients who underwent the study procedure are presented in Table 1.

**Figure 2:**
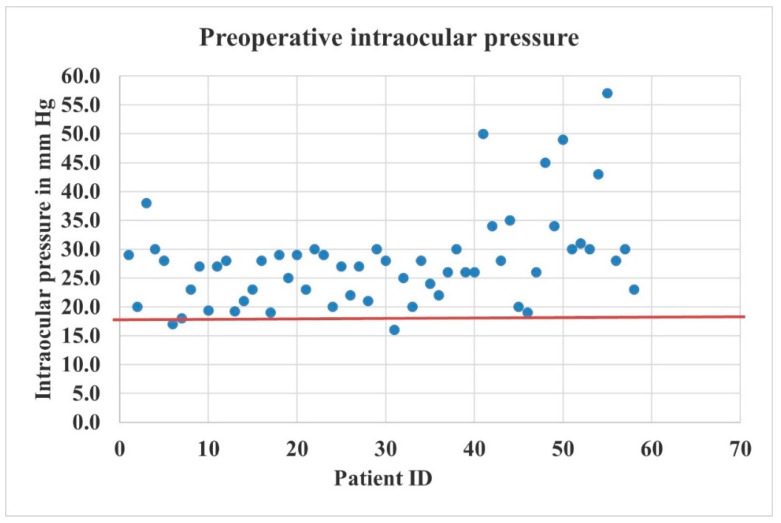
Scatterplot showing the distribution of preoperative IOP (n = 58 patients). The solid red horizontal line represents an IOP of 18 mmHg. Blue dot represents a case.

A scatterplot showing the distribution of preoperative IOP is presented in Figure 2.

### 3.1. IOP Change

**Figure 3:**
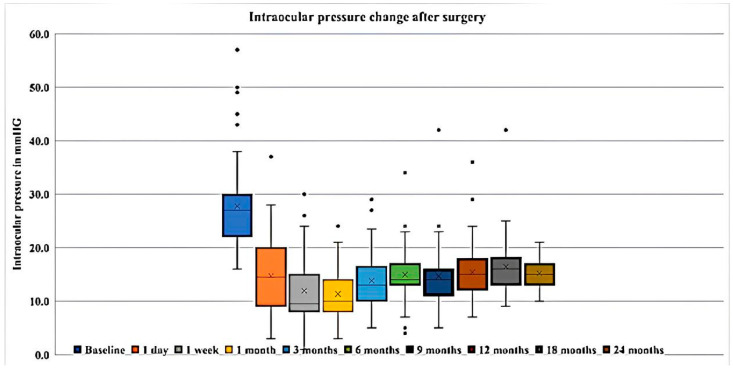
Box-and-whisker plot showing post-surgical IOP changes: The colored boxes represent the 25th and 75th percentiles; the solid horizontal lines in the colored boxes represent the median; X represents the mean; vertical solid black lines extend the interquartile range 1.5 times; the dots represent outliers.

**Figure 4:**
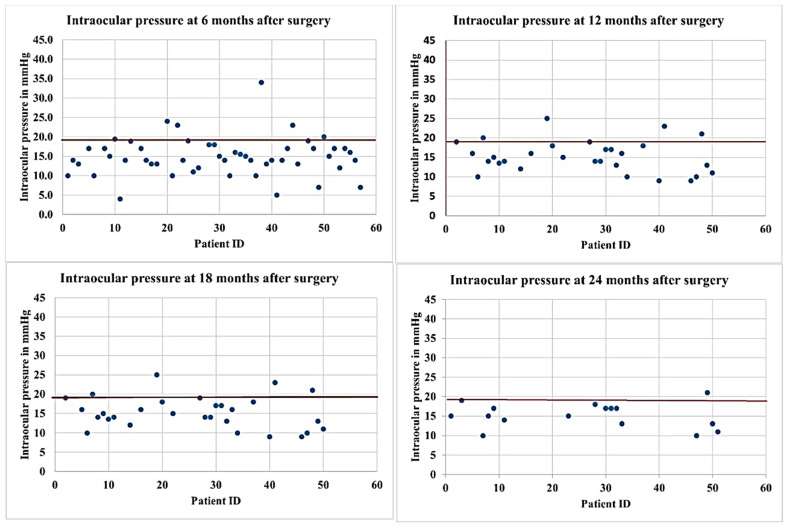
Scatterplots of IOP values at different follow-up visits. The solid horizontal lines represent the target IOP = 18 mmHg. A blue dot represents a case.

Following surgery at 6, 12, 18, and 24 months, the baseline IOP mean ± SD (95% confidence interval) decreased to 14.9 ± 5.0 (13.5–16.3), with medians of 15.0 (13.0; 18.0), 16.5 ± 6.2 (14.1–18.8), and 15.2 ± 3.3 (13.5–16.9) mm Hg, respectively. The IOP change, percentage reduction in IOP from preoperative values, and *p*-values at follow-up visits are presented in Table 2 and illustrated in Figure 3 and Figure 4.

The distribution of patients as a percentage reduction in IOP at different visits is shown in Table 3. As can be observed from the table, most patients presented a decrease in their IOP values by more than 40%.

### 3.2. Change in Use of Hypotensive Medications

**Figure 5:**
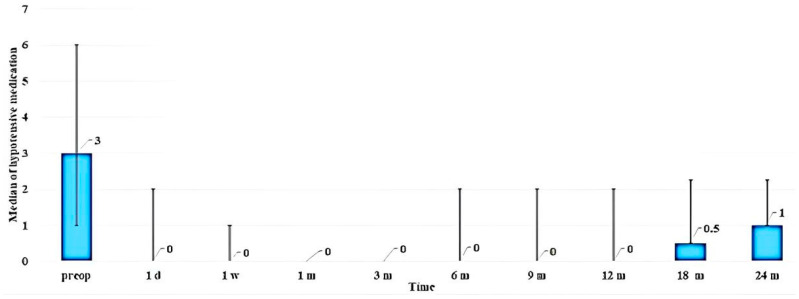
Bar diagram showing the median numbers of hypotensive medications used after surgery at different follow-up times.

The median number of ocular hypotensive medications used by patients preoperatively was 3 (2; 3). At 6, 12, and 24 months, the use of medications reduced to 0 (0; 2), 0 (0; 2), and 1 (0; 1.25), respectively. The change in the use of hypotensive medications following surgery is shown in Figure 5. As can be observed from the diagram, additional hypotensive medication was required by patients after periods of 18 and 24 months. 

### 3.3. Visual Acuity

**Figure 6:**
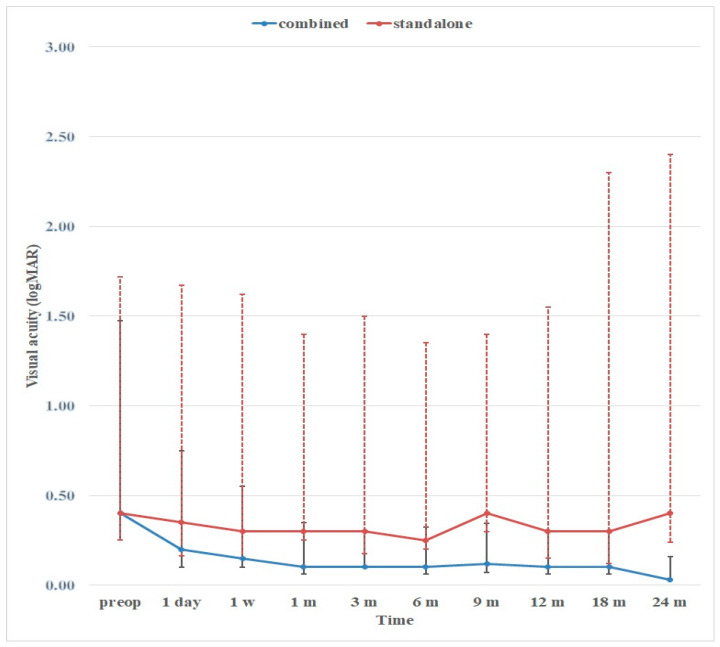
Preoperative versus postoperative logMAR VA (median) values for patients having undergone combined procedures (blue solid horizontal line showing the median VA and blue solid vertical lines showing p^25^ and p^75^) and glaucoma surgery as a standalone procedure (red solid horizontal line showing median VA and red broken vertical lines showing p^25^ and p^75^).

The average VA improved in all cases where a combined surgery was performed (Figure 6), whereas it remained unchanged in cases where glaucoma surgery was performed as a standalone procedure. 

### 3.4. Success

At 6, 12, 18, and 24 months, complete success was achieved in 62.8% (32/51), 51.2% (22/43), 46.7% (14/30), and 32.5% (6/16) of cases, respectively, and partial success was achieved in 28.4% (14/51), 44.2% (19/43), 50% (15/30), and 62.5% (10/16) of cases, respectively. Eight cases (13.8%) did not achieve target IOP values, of which five cases were declared as failures at 6 months, another two cases at 12 months, and another one case at 18 months. In four cases, the reason was the blockage of the trabeculotomy by iris tissue. Four additional cases presented advanced-stage glaucoma, of which three cases previously had undergone failed filtering surgeries.

**Figure 7:**
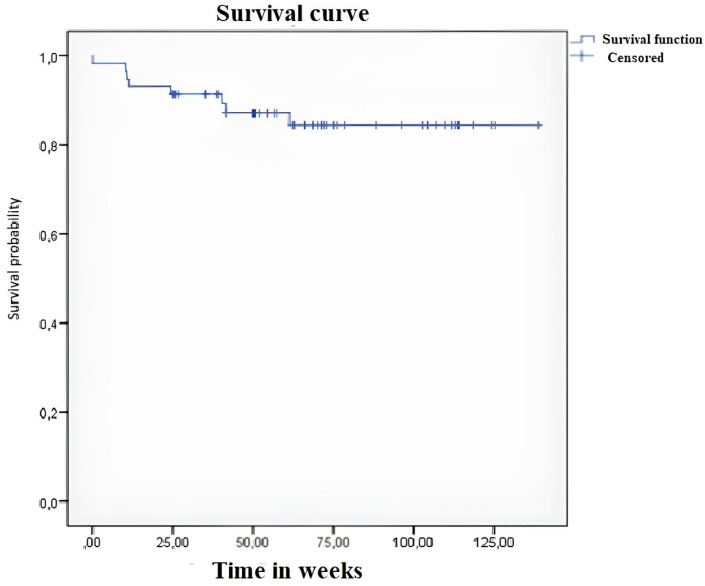
Kaplan–Meier survival curve after surgery.

A Kaplan–Meier curve showing the success of treatment is presented in Figure 7.

### 3.5. Observations during Surgery

In all 16 cases having previously undergone filtration surgeries, the surgery was attempted close to the previous surgical area, and certain difficulties in performing the dissection of the conjunctival and scleral flaps were faced by the surgeons because of existing fibrosis. However, no difficulties or complications were encountered during the operation in these cases while creating a supraciliary tunnel and inserting the CI into it. No complications related to glaucoma surgery were noticed in any of the other cases. 

### 3.6. Observations in the Postoperative Period

**Figure 8:**
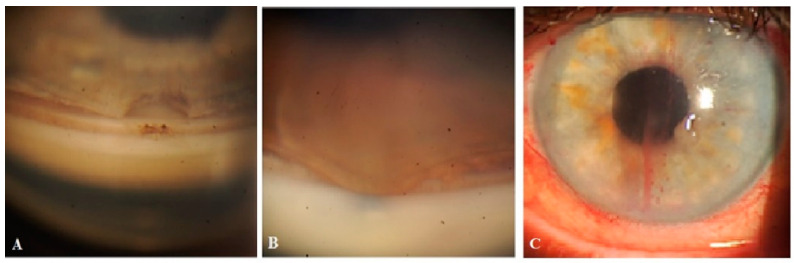
Complications after receiving Nd:YAG laser trabeculotomy: (**A**) Trabeculotomy blockage by pigment. (**B**) Blockage by iris tissue. (**C**) Blood oozing from Schlemm’s canal.

Postoperatively, one case had spontaneous retinal hemorrhage, which was not related to surgery and resolved after one month. There were no cases of hypotony or shallow AC during the observation period. Elevated IOP due to blockage of the trabeculotomy by iris tissue (Figure 8A,B) was observed in four cases; in two cases, the IOP was medically controlled, and in another case a repeat trabeculotomy was performed close to the blockage site. In the fourth case, a second surgery was required.

### 3.7. Nd:YAG Laser Trabeculotomy

Nd:YAG laser trabeculotomy was required in 67.2% of cases (39/58). Some oozing of blood from Schlemm’s canal after the procedure was observed in two cases (Figure 8C). The hyphema resolved spontaneously within one week. 

The distribution of patients according to the time that passed between the surgery and laser trabeculotomy is presented in Table 4. As can be seen from the table, most of the trabeculotomies were performed within one month following surgery—in 84.6% of cases (33 out of 39 eyes). 

Out of 39 eyes, trabeculotomy was performed once on 30 eyes; the other 9 eyes (23%) required more than one trabeculotomy procedure. Of these, in six eyes, the trabeculotomy was performed twice to achieve the target-level IOP, reaching the target level in only two eyes. Four additional eyes required additional hypotensive medications. In another two eyes, trabeculotomies were performed three times, and in one eye four times—all in vain. A second trabeculotomy was required in four eyes within a period of one month, in one eye after four months, and in another eye after seven months.

#### IOP Decrease after Nd:YAG Laser Trabeculotomy

The IOP decrease after the Nd:YAG laser trabeculotomy procedure is shown in Table 5. After the procedure, in 76.9% of eyes (30/39), the IOP values were less than 15 mmHg. 

During the Nd:YAG laser trabeculotomy procedure, the following events were observed: difficulty in identifying the surgical site—4 cases; pulsatile AH flow after trabeculotomy—12 cases; and blood oozing from trabeculotomy—2 cases. After trabeculotomy, in one case, the Tindal phenomenon was observed, requiring the instillation of anti-inflammatory medication (dexamethasone 0.1% eye drops; 1–2 drops three times daily) for 3–4 days. Elevated IOP due to blockage of the trabeculotomy by iris tissue was observed in four cases. To prevent this complication from occurring, in cases that underwent combined procedures, a peripheral iridotomy using a 100 µm tip of a Fugo plasma blade was performed at the end of cataract surgery. In standalone cases prior to surgery, Nd:YAG laser iridotomy was performed in the outpatient department. 

### 3.8. Filtration Bleb

Some tissue reaction to surgical trauma was noticed at the surgery site on the day after surgery. The OCT of the surgical site demonstrated the absence of blebs in all cases except one, where a bleb lasted for a period of one month.

### 3.9. CLV Development

The postoperative development of lymphatic vessels in the conjunctiva was observed in 50% of cases (29/58). Biomicroscopically, these vessels had uneven calibers and were visible in the form of a plexus or solitary vessels running parallel to the limbus or perpendicular to it. The lymphatic nature was confirmed by OCT [21]. Sausage-type vessels with valve-like structures (VLSs) in their lumens were evident. 

The time of CLV development differed from case to case. In 6 eyes, CLVs developed the following day after Nd:YAG laser trabeculotomy was performed, in 7 eyes after one week, and in 14 eyes after one month. In summary, in 93.1% of eyes (27 out of 29 eyes), CLVs developed within one month. In two eyes, CLVs developed late, at six months in one eye and at nine months in another eye. 

The CLVs lasted for different periods of time. The time of CLV development and the duration of CLV visibility via biomicroscopy are presented in Table 6. On OCT, CLVs with VLSs in their lumens were diagnosed in most cases—in 82.8% of eyes (48/58). 

**Figure 9:**
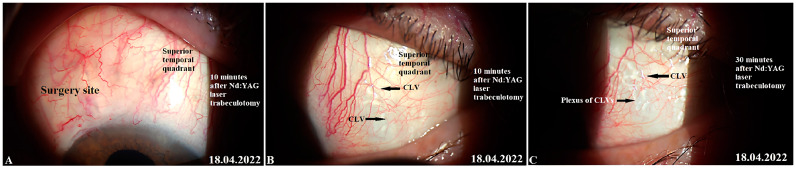
Slit-lamp view of the surgical site and superior quadrants in a patient’s left eye in which CLVs developed immediately after Nd:YAG laser trabeculotomy: (**A**) Ten minutes after Nd:YAG laser trabeculotomy, the surgical site and nearby areas are free from CLVs; some swelling at the surgical site persists. (**B**) Ten minutes after Nd:YAG laser trabeculotomy, another slit-lamp view of the superior temporal quadrant showing a plexus of CLVs (black arrows). (**C**) Thirty minutes after the procedure, slit-lamp view of the superior temporal quadrant after massaging the area with the lid margin; a well-developed plexus of CLVs can be observed far away from the surgery site. CLV = conjunctival lymphatic vessel; Nd:YAG laser = neodymium yttrium aluminum garnet laser.

**Figure 10:**
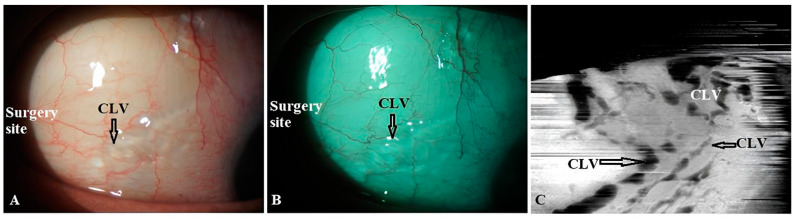
Slit-lamp view of the right eye of a patient in whom CLVs developed without Nd:YAG laser trabeculotomy: (**A**) Slit-lamp view of the superior temporal quadrant of the eyeball on day 21 after surgery, showing a plexus of CLVs developed far away from the surgery site (arrow with black borders). (**B**) The same view with a red-free filter. (**C**) Same site as in (**A**,**B**); an Enface image (OCT-angio) demonstrating sausage-shaped lymphatic vessels with uneven calibers (arrows with black borders). CLV = conjunctival lymphatic vessel.

No relationship was established between Nd:YAG laser trabeculotomy and the development of CLVs. CLVs were observed to develop irrespective of Nd:YAG laser trabeculotomy (Figure 9 and Figure 10). Of thirty-nine eyes that underwent Nd:YAG laser trabeculotomy, CLVs developed in 46.1% of eyes (18/39 eyes), whereas in eyes without laser trabeculotomy they developed in 52.6% of eyes (10/19 eyes), suggesting that the role of laser trabeculotomy was limited to providing resistance-free AH flow from the AC to the SCS. 

Interesting facts were uncovered when the site of CLVs’ development was analyzed as per the operated eye. The results are presented in Table 7. In cases where surgery was performed on the right eye, CLVs developed more commonly in the superior nasal quadrant, and in cases where surgery was performed on the left eye, CLVs developed more commonly in the superior temporal quadrant. Out of 17 cases where glaucoma surgery was performed on the right eyes of patients, in 16 eyes (94.1%) CLVs developed in the superior nasal quadrant. Of these, in five eyes, CLVs developed in both quadrants. The quantitative analysis of CLVs identified on OCT presented the same pattern—in the superior nasal quadrant, they were identified in 20 out of 24 eyes (83.3%). Nearly the same pattern of CLV development was noticed in the left eyes of patients. Out of 12 eyes, CLVs were predominant in the superior temporal area in 9 eyes (75.0%). The same was true for OCT—CLVs were identified in the superior temporal area in 60.7% of eyes (17/28). 

In all except one case, CLVs developed in an area located far away from the surgical site. CLVs developed directly from the sclera, indicating the existence of intrascleral microchannels located across the sclera connecting the SCS to the CLVs (Figure 9C). Clinically, we observed a few patients in whom CLVs first appeared in one quadrant; with time, they disappeared, but new CLVs developed in another quadrant (refer to case 2). 

To define the relationship between CLV development and the decrease in IOP, all cases were divided into two subgroups: subgroup I included cases with CLVs identified on slit-lamp examination, and subgroup II included cases without CLV identification. In each subgroup, the success rate was analyzed. The results are presented in Table 8. The number of cases in both subgroups was equal. The analysis showed that the rate of complete success was higher in subgroup II, achieving 75.9% versus 37.9% in subgroup I (*p* < 0.001). The qualified success rate was equal in both subgroups. The instillation of hypotensive medications to achieve target IOP values was required by more patients in subgroup I—by 44.8% patients versus 13.8% patients in subgroup II (*p* < 0.0001). There was no difference in failure rates between the two subgroups. These results suggest that the development of CLVs after surgery has a poor prognostic value for IOP control. If the fluid flow from the SCS to CLVs through intrascleral microchannels is smooth and resistance-free, no CLV development is evident. However, if any resistance exists in the flow, the fluid accumulates in lymphatics, resulting in their engorgement and a longer visibility period.

Below, we present a few clinical cases to demonstrate the effectiveness and safety of the proposed technique in decreasing IOP, the role of Nd:YAG laser trabeculotomy, the impact of CLV development on IOP decrease, and the possible mechanism of the hypotensive effect produced by the technique without forming a filtration bleb. Relevant demographic and clinical features of three cases having undergone combined procedures for coexisting pathologies are provided in Supplementary Table S1.

### 3.10. Case Reports

Case 1: Patient K, a 78-year-old female patient, underwent combined surgery in both eyes. Glaucoma was diagnosed five years ago and, since then, the patient has been on four classes of hypotensive medications in both eyes (analog of prostaglandins F2α, beta-adrenergic blocker, carbonic anhydrase inhibitor, and selective α 2-adrenomimetic). Her IOP prior to surgery was 27.0 mmHg in both eyes. The patient had a history of intravitreal injections of anti-VEGF (sol. Lucentis—three injections) for wet macular degeneration in her right eye. 

After surgery, the patient underwent Nd:YAG laser trabeculotomy in the operated area in both eyes. The IOP in her right and left eyes, at 1 week and at 1, 3, 6, 12, 18, and 24 months, was 13, 13, 9, 11, 15, and 15 mm Hg and 6, 10, 10, 15, 10, and 10 mm Hg, respectively, off medication. The absence of blebs was confirmed by OCT at all follow-up visits. 

**Figure 11:**
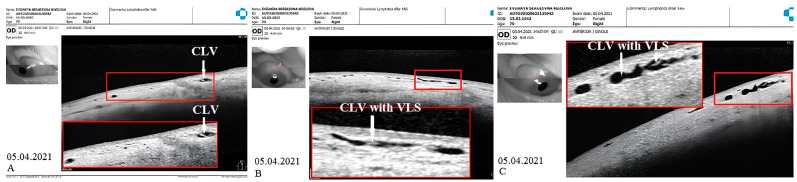
Case 1 (right eye): Follow-up visit at 12 months. OCT scans of the (**A**) superior nasal quadrant, (**B**) surgical site, and (**C**) superior temporal quadrant showing a CLV with a VLS in its lumen (white arrow). Enlarged scans of the area of interest are displayed nearby, enclosed by a red boundary. CLV = conjunctival lymphatic vessel; VLS = valve-like structure.

**Figure 12:**
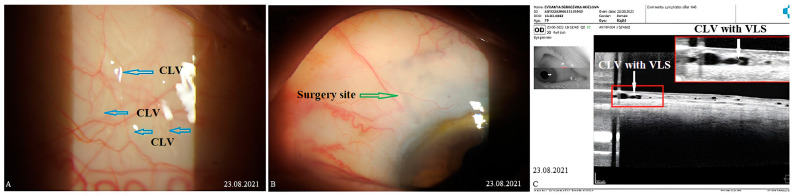
Case 1 (right eye): Follow-up visit at 15 months. (**A**) Slit-lamp view of the superior nasal quadrant showing a few CLVs, which are located far from the surgical site, and which arose directly from sclera (arrow with sky-blue border). (**B**) Slit-lamp image of the superior temporal quadrant and surgical site, showing the absence of blebs and CLVs (arrow with green border). (**C**) OCT scan of the superior nasal quadrant showing a CLV with a VLS in its lumen (white arrows); an enlarged scan of the area of interest is displayed nearby, enclosed by a red boundary. CLV = conjunctival lymphatic vessel; VLS = valve-like structure.

**Figure 13:**
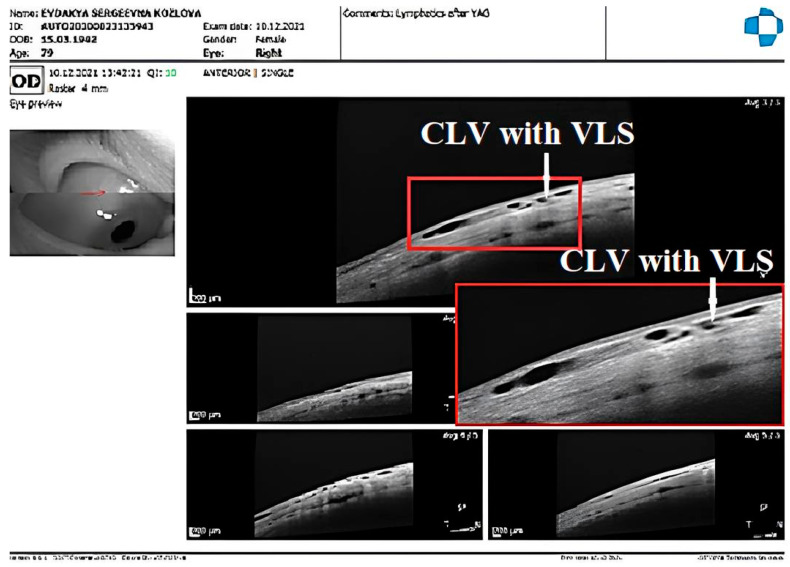
Case 1 (right eye): Follow-up visit at 18 months. OCT horizontal scan of the superior temporal quadrant showing a CLV with a VLS (white arrows). An enlarged image of the area of interest is presented. CLV = conjunctival lymphatic vessel; VLS = valve-like structure.

CLV development in the right eye: The following day after the trabeculotomy, a well-developed plexus of CLVs was identified biomicroscopically in the superior nasal quadrant. With the passage of time, these vessels reduced in number and size (Figure 11, Figure 12 and Figure 13). At the last follow-up visit, at 2.5 years, no CLVs were identified biomicroscopically. However, on OCT, CLVs were identifiable in the nasal and temporal quadrants until the last follow-up visit.

**Figure 14:**
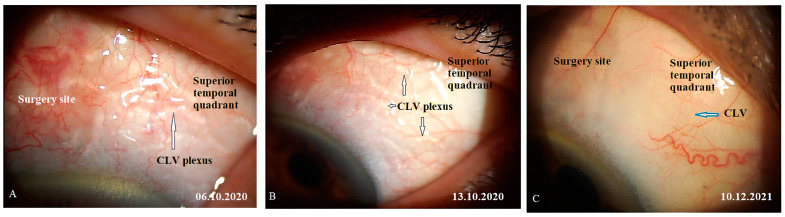
Case 1 (left eye): CLV development after surgery. (**A**) Slit-lamp view of the superior temporal quadrant on the day after laser trabeculotomy; a plexus of engorged CLVs with uneven calibers and filled with transparent fluid; some of these vessels originated from the surgical site, and most of them run parallel to the limbus. (**B**) Slit-lamp view of the superior temporal quadrant after 1 week; the size and number of vessels are considerably reduced, and they are less engorged. (**C**) Slit-lamp view of the superior temporal quadrant at follow-up visit at 14 months; an isolated CLV originating directly from the sclera located far from the surgical site; at the surgical site, there is no bleb or CLV. CLV = conjunctival lymphatic vessel; VLS = valve-like structure.

**Figure 15:**
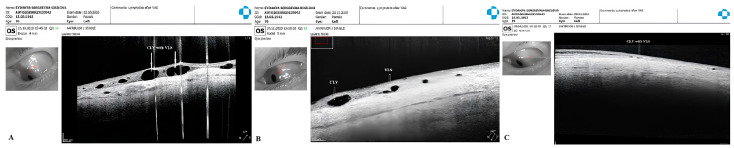
Case 1 (left eye): CLV dynamics upon OCT investigation at different follow-up visits. (**A**) OCT scan of the superior temporal quadrant 2 weeks after laser trabeculotomy, showing well-developed CLVs with VLSs in their lumens. (**B**) OCT scan of the same area as A; a CLV with a VLS in its lumen at 4 weeks; the vessel is reduced in size. (**C**) OCT scan of the same areas in A and B, at 6 months, showing a CLV with a VLS in the lumen. The vessel is nearly unidentifiable. CLV = conjunctival lymphatic vessel; VLS = valve-like structure.

**Figure 16:**
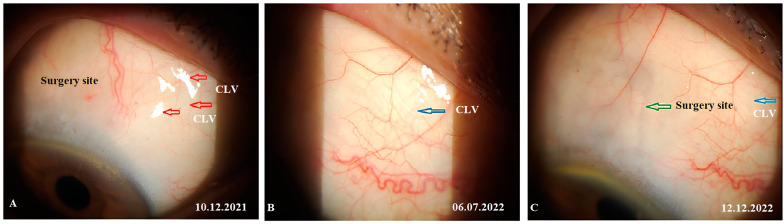
Case 1 (left eye): Slit-lamp images of late follow-up visits. (**A**) Slit-lamp view of the superior temporal quadrant and surgical site showing the absence of blebs and a few questionable isolated CLVs (arrow with red border) at the follow-up visit at 14 months. (**B**) Slit-lamp view of the superior temporal quadrant at 18 months and a few collapsed CLVs (arrow with blue border). (**C**) Slit-lamp view at 24 months after surgery. No bleb is observed at the surgery site (arrow with green border), and there is an isolated CLV in the temporal quadrant (arrow with sky-blue border). CLV = conjunctival lymphatic vessel.

CLV development in the left eye: Like in the right eye, in the left eye on the day after the laser trabeculotomy, a plexus of CLVs was also observed in the superior temporal quadrant (Figure 14A). During the subsequent follow-up visit, one week after the trabeculotomy, the CLVs were reduced in size and number (Figure 14B). By month 14, only an isolated CLV could be observed in the temporal quadrant (Figure 14C). The surgical site and superior nasal and temporal quadrants, which were investigated by OCT, showed CLVs with VLSs in their lumens (Figure 15A–C). At the final follow-up visit, CLVs were not visible biomicroscopically (Figure 16A–C).

The role of Nd:YAG laser trabeculotomy in providing the resistance-free flow of AH from the AC to the SCS was emphasized in this case. The patient underwent combined surgery and laser trabeculotomies after surgery on both eyes. Prior to the trabeculotomy, sufficient time was allowed for the conjunctiva to heal, thus preventing AH flow to the subconjunctival space, as shown by OCT. Until the last follow-up visit, the patient presented a significant decrease in IOP without a filtering bleb. Postoperatively, CLVs, which were identified biomicroscopically, developed in both eyes. Notably, once the proper AH flow from the SCS to the CLVs was established, these vessels reduced in size and number, leaving a few (identified on OCT) to maintain the flow. 

Case 2: Patient R, an 80-year-old male patient, was operated upon on his left eye. The patient had glaucoma in both of his eyes, which was diagnosed seven years prior and, since then, he has been on two classes of hypotensive medication (a combination of a carbonic anhydrase inhibitor and a beta-adrenergic blocker). For demographic and clinical features, please refer to Supplementary Table S1. The surgery went uneventfully. Conjunctival sutures were removed on day 10. His IOP was 10 mmHg on this day, and he was advised to discontinue the instillation of hypotensive medication. As the patient’s IOP was below the target level at all visits, no Nd:YAG laser trabeculotomy was performed. Postoperative logMAR VA improved to 0 and remained at this value until the final follow-up visit at 12 months. 

**Figure 17:**
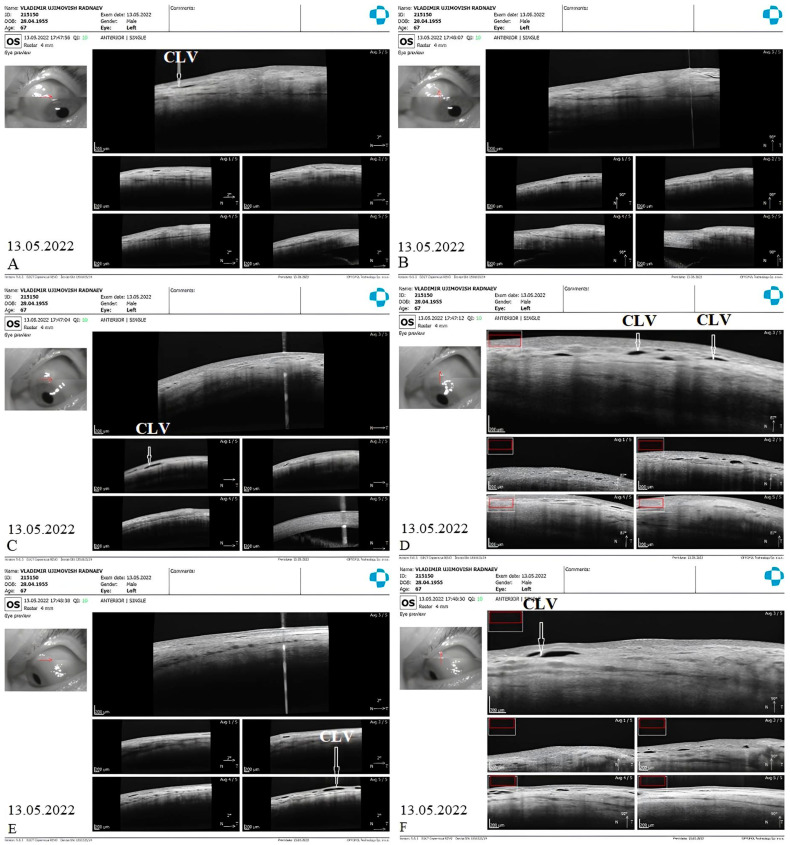
Case 2 (left eye): OCT evaluation of the surgical site and superior quadrants 1 week after surgery: horizontal (**A**) and vertical (**B**) scans of the superior nasal quadrant showing a CLV on the horizontal scan (arrow with white border); horizontal (**C**) and vertical (**D**) scans of the surgical site showing CLVs (arrows with white borders); horizontal (**E**) and vertical (**F**) scans of the superior temporal quadrant showing CLVs (arrow with white border) with VLSs in their lumens. CLV = conjunctival lymphatic vessel.

CLV development: At one week after surgery, slit-lamp biomicroscopy revealed an absence of blebs and CLVs. On OCT, the absence of a bleb at the surgical site was confirmed, and CLVs were identified in the superior nasal and temporal quadrants, as well as at the surgical site (Figure 17A–F).

**Figure 18:**
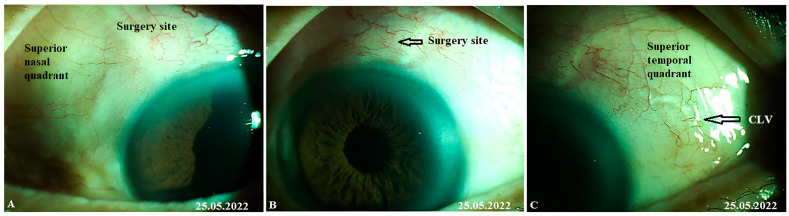
Case 2 (left eye): Slit-lamp view (red-free filter) at 1 month after surgery. (**A**) Slit-lamp view of the superior nasal quadrant, showing the absence of any CLVs. (**B**) Slit-lamp view of the surgical site, demonstrating the absence of blebs and CLVs (arrow with black border). (**C**) Slit-lamp view of the superior temporal quadrant showing CLVs (arrow with black border); CLVs have no connection to the surgical site; they arise directly from the sclera, located at a considerable distance from the surgical site. CLV = conjunctival lymphatic vessel.

The patient was then consulted after another 2 weeks. On biomicroscopy, CLVs were observed in the superior temporal quadrant (Figure 18A–C); the lymphatics arose directly from the sclera, located at a considerable distance from the surgical site, and became more prominent when the upper eyelid was moved over them (see Supplementary Materials, Video S1_Case 2_month 1 after surgery: CLVs in superior temporal quadrant).

**Figure 19:**
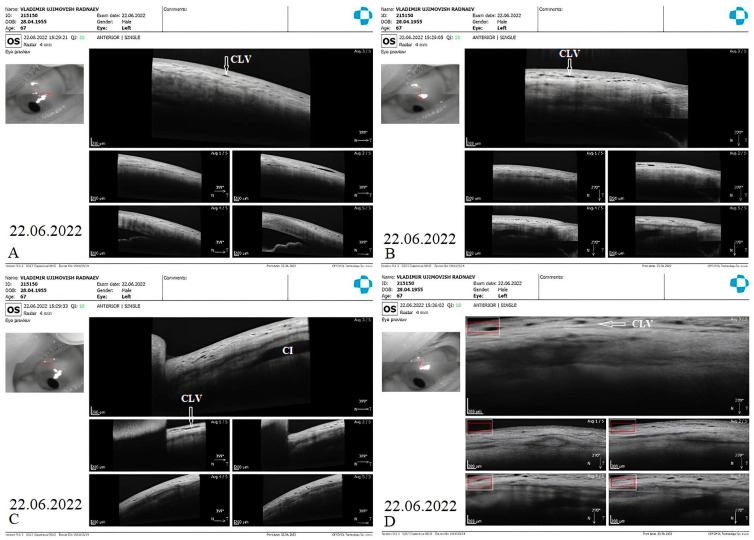
Case 2 (left eye): OCT investigation at 1.5 months after surgery. OCT raster scans of the (**A**,**B**) superior temporal quadrant and (**C**,**D**) surgical site, demonstrating CLVs (arrows with white borders); in scan C, a collagen implant (CI) can also be observed in the ISR, and there is no bleb above it. Scans of the superior nasal quadrant are not included, as they lack any useful information. CLV = conjunctival lymphatic vessel; CI = collagen implant.

The patient’s subsequent consultation was 1.5 months after surgery. Remnants of CLVs could be identified in the superior temporal quadrant on slit-lamp examination. On OCT, CLVs were identifiable in the superior temporal quadrant and at the surgical site (Figure 19A–D).

**Figure 20:**
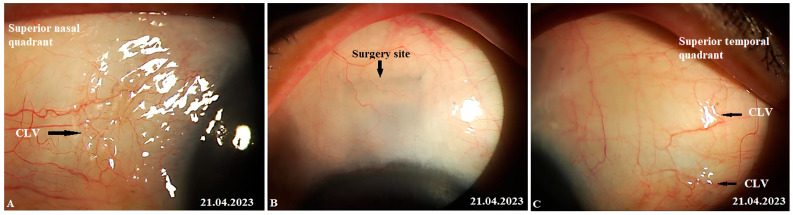
Case 2 (left eye): Slit-lamp views at 12 months after surgery. (**A**) Slit-lamp view of the superior nasal quadrant showing a well-developed plexus of CLVs (black horizontal arrow); the vessels became engorged after a gentle massage moving the eyelid over them (refer to Video S2_Case 2_month 12 after surgery); CLVs show no connection with the surgical site; they arise directly from the sclera located at a considerable distance from the surgical site. (**B**) Slit-lamp view of the surgical site demonstrating the absence of filtration blebs and CLVs. (**C**) Slit-lamp view of the superior temporal quadrant showing remnants of a previously developed plexus of CLVs (black arrow). CLV = conjunctival lymphatic vessel.

At 12 months, no bleb was noticed at the surgical site and, notably, no CLVs were observed in the superior temporal quadrant; instead, close to the insertion of the internal rectus muscle and in the superior nasal quadrant, a plexus of CLVs was observed. Massaging the conjunctiva over the plexus by moving the lid margin over it caused the vessels to become engorged (Figure 20A–C). Please refer to Supplementary Materials, Video S2_Case 2_month 12 after surgery: CLVs in superior nasal quadrant.

By reporting this case, we aimed to emphasize the effectiveness of the proposed technique in decreasing the IOP level without performing Nd:YAG laser trabeculotomy. The deroofing of the SC and the thinning of its inner wall by removing a part of the JCT was sufficient for AH outflow from the AC to decrease IOP significantly. This case also emphasized that the development of CLVs occurs irrespective of laser trabeculotomy. The deroofed SC and its thinned inner wall allowed enough AH to flow from the AC to the SCS to initiate its outflow across the sclera to CLVs. AH flowed across the sclera through some pores or microchannels. Another important observation that we made in this case was that CLVs first appeared in the superior temporal quadrant and lasted for a period of 12 months. Towards the end of this observation period, another plexus of CLVs developed in the superior nasal quadrant. This meant that as soon as the existing lymphatic pathway ceased to be functional for some reason, the CLVs developed in another area where a functional natural outflow pathway from the SCS to CLVs remained. For practical purposes, this case emphasizes that it is paramount to preserve the lymphatics while performing glaucoma surgery.

**Figure 21:**
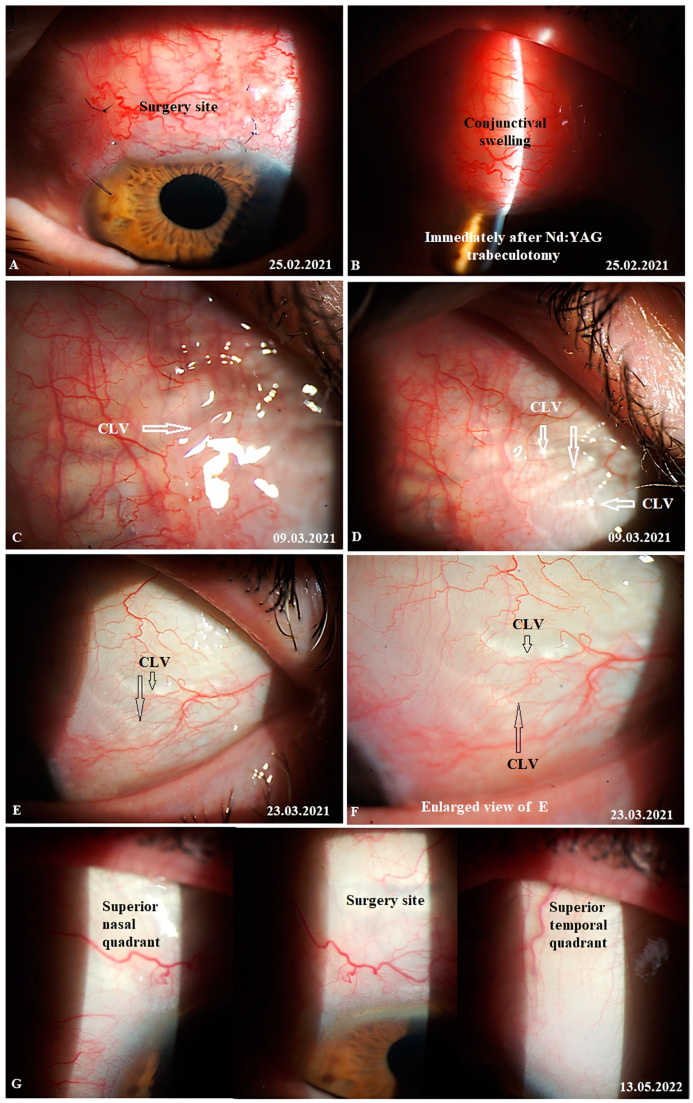
Case 3 (left eye): Postoperative follow-up visits. (**A**) Slit-lamp view of the surgical site before sutures were taken out. There is no bleb at the surgery site. (**B**) Slit-lamp view of the surgical site after sutures were removed and Nd:YAG laser trabeculotomy was performed, resulting in conjunctival swelling at the surgical site. OCT confirmed the absence of a bleb. (**C**,**D**) Slit-lamp view of the surgical site and superior temporal quadrant 12 days after trabeculotomy; a plexus of engorged CLVs was observed at the surgical site (arrows with white borders); its course could be detected towards the superior temporal quadrant, and no CLVs were visible in the superior nasal quadrant. (**E**,**F**) Slit-lamp view of the superior temporal quadrant after another 2 weeks; the CLV plexus disappeared from the surgical site, leaving few CLVs in the superior temporal quadrant (arrows with black borders) with reduced sizes. (**G**) Slit-lamp view of the surgical site and superior nasal and temporal quadrants 15 months after the postoperative follow-up visit. No blebs or CLVs could be detected. CLV = conjunctival lymphatic vessel.

Case 3: Patient A was a 69-year-old female patient who underwent a combined procedure for a hypermature cataract and advanced-stage OAG in her left eye. Glaucoma was diagnosed eight years prior and, since then, the patient has been on hypotensive medications (for demographic and clinical features, please refer to Supplementary Table S1). The surgery was uneventful. On day 12, the conjunctival sutures were removed (Figure 21A).

IOP changes: On day 12, the patient’s IOP on medication was 18 mmHg. On the same day, Nd:YAG laser trabeculotomy was performed. Immediately after the trabeculotomy, the patient’s IOP decreased to 16 mmHg and the patient was advised to stop the instillation of hypotensive medications. On the following two follow-up visits, the patient had elevated IOP levels up to 26 mmHg. Repeat-laser trabeculotomy was attempted two more times, with an interval of 7 days. After the third trabeculotomy attempt, the patient’s IOP decreased to 7 mmHg off medication. The IOP remained below the target level until 3 months, when an increase in the IOP to 31 mmHg was noticed. A fourth YAG trabeculotomy was attempted to decrease the IOP; however, this failed. The patient was advised to restart the instillation of hypotensive medications. At 6 and 12 months, the patient’s IOP levels were 34 and 24 mmHg, respectively, on three classes of hypotensive medications. A repeat glaucoma surgery was advised and a diode-laser cyclophotodestruction procedure was performed.

**Figure 22:**
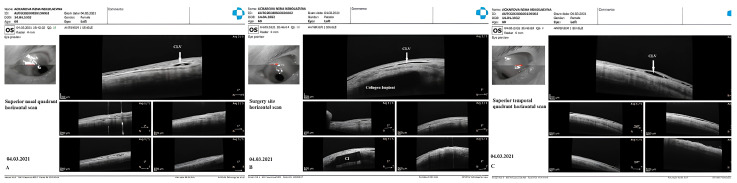
Case 3 (left eye): OCT investigation at 1 week after trabeculotomy. OCT scans of the (**A**) superior nasal quadrant, (**B**) surgical site, and (**C**) superior temporal quadrant. CLVs with valve-like structures could be detected at all investigated sites (white arrows); in (**B**), the collagen implant is clearly visible without any blebs over it. CLV = conjunctival lymphatic vessel.

**Figure 23:**
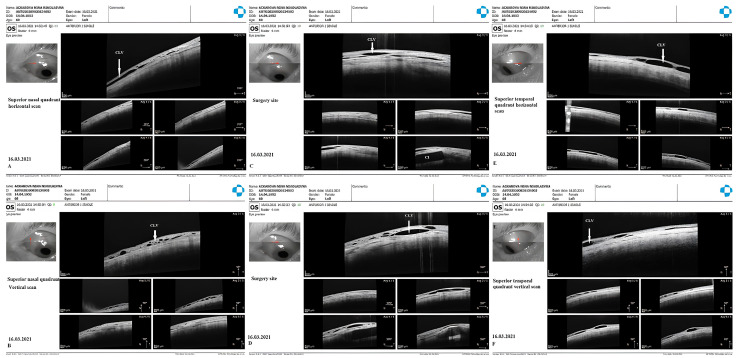
Case 3 (left eye): OCT investigation at 3 weeks after trabeculotomy. OCT raster scans of conjunctiva in the superior nasal quadrant ((**A**): horizontal scan, (**B**): vertical scan) over the surgical site ((**C**): horizontal scan, (**D**): vertical scan) and superior temporal quadrant ((**E**): horizontal scan, (**F**): vertical scan); CLVs with valve-like structures could be identified in all investigated areas (white arrows). CLV = conjunctival lymphatic vessel.

**Figure 24:**
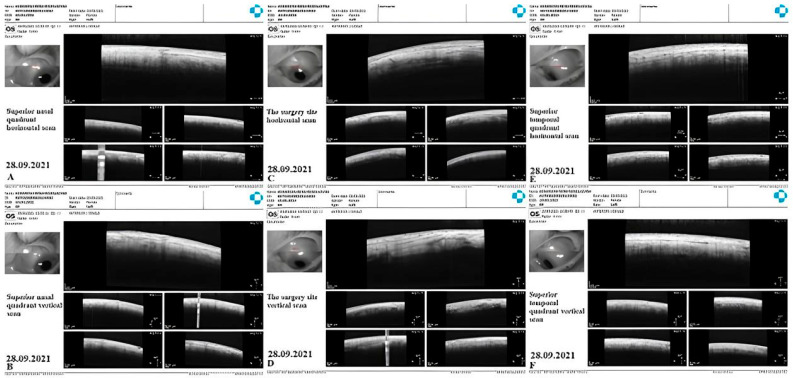
Case 3 (left eye): OCT investigation at 7 months after surgery. OCT raster scans of the conjunctiva in the superior nasal ((**A**): horizontal scan, (**B**): vertical scan) and temporal quadrants ((**E**): horizontal scan, (**F**): vertical scan) and at the surgical sites ((**C**): horizontal scan, (**D**): vertical scan); no blebs or CLVs could be identified in any of the investigated areas.

**Figure 25:**
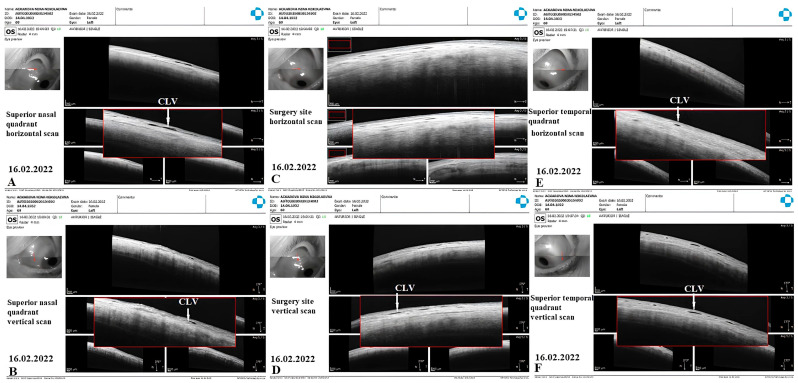
Case 3 (left eye): OCT investigation at 12 months after surgery. OCT raster scans of conjunctiva in the superior nasal ((**A**): horizontal scan, (**B**): vertical scan) and temporal quadrants ((**E**): horizontal scan, (**F**): vertical scan) and at the surgical sites ((**C**): horizontal scan, (**D**): vertical scan); a few CLVs with valve-like structures in their lumens could be identified in all investigated areas. CLV = conjunctival lymphatic vessel.

CLV development: Immediately after the first Nd:YAG laser trabeculotomy, some conjunctival swelling appeared at the surgical site (Figure 21B). After 1 week, the slit-lamp images showed overfilled CLVs with uneven calibers in the form of a plexus, which became visible at the surgical site (Figure 21C). Their course from the surgical site could be detected towards the superior temporal quadrant (Figure 21D). CLVs remained visible biomicroscopically for up to 2 months after the trabeculotomy. With time, their number and size decreased considerably (Figure 21E,F). At 7 months, the CLVs completely disappeared (Figure 21G). At each follow-up visit, the patient’s surgical site and nearby quadrants were evaluated by OCT (Figure 22, Figure 23, Figure 24 and Figure 25). CLVs with VLSs were present in the conjunctiva over the surgical site and in both superior quadrants at all follow-up visits.

This case demonstrated the activation of AH flow from the SCS to CLVs after Nd:YAG laser trabeculotomy was performed. Like the previous cases, outflow activation occurred without the formation of a filtering bleb. The appearance of engorged CLVs over the surgical site and in the superior temporal quadrant within a period of 7 days after laser trabeculotomy indicated that, with the proposed technique, the functional status of conjunctival lymphatics played a considerable role in IOP regulation. Overfilled engorged CLVs highlighted the possible resistance in the fluid pathway or the unsatisfactory functional status of the conjunctival lymphatic system. This case emphasizes the necessity for early surgical intervention in glaucoma patients when conjunctival lymphatics are still active and functional.

## 4. Discussion

After traditional glaucoma surgeries, AH accumulates in a subconjunctival space (bleb). The long-term survival of the bleb is paramount to achieve a long-term hypotensive effect. Various modifications in surgical techniques, the use of different devices, and the peri- and postoperative use of antimetabolites have been proposed in the literature to achieve the long-term survival of blebs [24,25,26]. The use of antimetabolites causes a long-term reduction in conjunctival and lymphatic vessels in the bleb and surrounding tissue [27].

From blebs, AH filters through conjunctival vessels and lymphatics [6,7,28,29,30,31,32]. Benedikt O. [33,34] was the pioneer, who investigated AH drainage from a bleb after filtering surgery using fluorescence photography after an intracameral injection of fluorescein. From his observations, he claimed that if the tension was too low at the surgical site, in most cases, a filtering bleb would form. If the IOP was higher than the episcleral vein pressure, new vessels could develop and drain the AH from the scleral fistula, which meant that there would be a good pressure-regulating effect without a bleb.

Mini-invasive glaucoma surgeries (MIGSs) have been developed in the field to overcome the drawbacks of filtering glaucoma surgeries. These surgeries restore or enhance AH outflow through natural outflow pathways. At present, most of the surgical techniques developed in this direction aim at activating or enhancing AH outflow through the traditional trabecular outflow pathway [35,36,37,38]. These techniques have limited success, as they mostly address the resistance at the distal end of the trabecular outflow pathway, leaving resistance proximal to the posterior wall of Schlemm’s canal unattended [39,40,41].

Owing to the large surface area of a choroid, with its high resorptive capacity and large area of sclera overlying the choroid with its pores and preformed microchannels, which are capable of draining fluid directly into scleral veins, the non-trabecular pathway has tremendous potential to decrease IOP levels [42,43]. This pathway has a system of interconnected spaces, namely, intertrabecular fissures of the uveal layers of the trabecular reticulum, spaces between the bundles of ciliary muscle myocytes, SCSs with a valve-like suprachoroidal system, and paravasal spaces of trans-scleral vessels, which ensure the continuous movement of fluid from the AC along the vascular tract to the posterior parts of the eyeball [44]. Previously, surgeons accessed this route by performing surgical cyclodialysis. In cyclodialysis, the ciliary body is detached from the scleral spur, providing resistance-free AH access to the SCS. This procedure decreases the IOP significantly; however, its results are unpredictable. Its success rate solely depends upon the patency of the cyclodialysis tunnel. The detachment of the ciliary body from the scleral spur is traumatic, and cyclodialysis clefts have the tendency to close down due to the associated inflammation. Various modifications of the cyclodialysis procedure with implantations of different kinds of drainage devices and implants have been proposed in the research and are being used with variable success [14,16,45,46,47,48,49,50,51,52,53]. CyPASS, a device inserted in the cyclodialysis tunnel ab interno [54], showed promising results in terms of decreasing IOP; however, the long-term observations demonstrated a progressive loss of endothelial cells. As a result, this device was withdrawn from the market by the manufacturer [15]. Its implantation may result in a high incidence of sudden IOP peaks with a low success rate [55]. Several other devices have been proposed but are still under study [56,57].

Different techniques have been popularized to reroute AH flow from the AC to the SCS without performing surgical cyclodialysis [11,58]. Jordon et al. [11] proposed the insertion of a silicone tube as an intrascleral connection from the AC to the SCS and reported satisfactory surgical outcomes in 31 eyes of 31 patients with uncontrollable refractory glaucoma. According to the authors, the intrascleral course of the tube minimized the risk of conjunctival erosion and associated infections, and there was no need to perform cyclodialysis. Identical to the technique described by Jordon et al. [11], Unal M. et al. [58] proposed a technique in which a suprachoroidal silicone tube shunt was implanted after making a deep sclerotomy adjacent to the scleral flap’s opening; the posterior end of the silicone tube was placed posteriorly in the SCS, and the anterior end was placed into the AC. The authors reported satisfactory qualified success rates of 95.8% in the first week and 87.5% in the first, third, sixth, and twelfth months. Complications were few. No infection, nor any choroidal or retinal detachment, was observed. Failure was observed in seven eyes, of which three underwent reoperations for glaucoma.

The non-trabecular outflow pathway has been exploited by surgeons to enhance the hypotensive effect of other surgical procedures for the treatment of glaucoma. Dada et al. [59] combined trabeculectomy with a limited deep sclerectomy and cyclodialysis in two pseudophakic patients who developed secondary glaucoma after vitreoretinal surgery with silicone oil insertions. In this technique, the authors used excised scleral tissue obtained after performing a deep sclerectomy as a spacer to maintain the patency of the cyclodialysis cleft.

The intrascleral implantation of biocompatible CI was recommended by several authors to enhance the success rate of the non-penetrating deep sclerectomy procedure, and to reduce the effect of fibrosis at the surgical site [60,61,62]. These implants acted as space maintainers in the scleral bed and acted like a sponge, transporting the liquid via capillary action. Shaarawy T. et al. [60] conducted a comparative study between deep sclerectomies with and without CI, and they reported that deep sclerectomy augmented with the suprachoroidal implantation of a CI was more effective in the long-term lowering of IOP than with intrascleral CI [63]. Pershin K. et al. [64] also compared the results of deep sclerectomy and the implantation of CI in the surgical treatment of glaucoma and observed that, except for the lower rate of goniopuncture among patients with suprachoroidal implantations of CI, the results were comparable with those from patients in whom implants were placed intrasclerally.

Szurman P. et al. [65] modified the canaloplasty technique to maximize the IOP-lowering effect by altering both the trabecular and non-trabecular aqueous outflow rates. The authors reduced full-thickness deep scleral layers to the choroid, opening the SCS. The authors retrospectively analyzed the surgical outcomes of 78 eyes and showed a significant decrease in mean IOP values from 19.1 mmHg on three topical medications to 13.5 mmHg on one topical medication at twelve months.

In the present study, the medium- and long-term results of a technique to enhance AH outflow from the AC to the SCS without detaching the ciliary body from the scleral spur are presented. With the technique described above, AH, after leaving the AC, accumulated in the ISR and, maintaining a physiological pressure, entered the SCS, from where it outflowed through uveoscleral, uveovortex, and uveolymphatic routes. Clinical observations and the results of OCT investigations achieved in this study—namely, a decrease in IOP without forming a filtering bleb, the activation of fluid flow from the SCS to CLVs, and CLV development—indicate that some of the AH flow from the SCS occurred via intrascleral microchannels to CLVs. This route may be referred to as the *SCS–intrascleral microchannels–CLVs* route.

In the literature, we did not observe CLV developments following the enhancement of AH flow to the SCS. To the best of our knowledge, this is the second report to mention the development of CLVs. Previously, we reported the results of a pilot study consisting of 38 patients in whom glaucoma surgery was performed using the technique mentioned above [18]. In that study, at six and twelve months, a significant decrease in IOP levels without forming a filtering bleb, along with a reduction in the use of additional hypotensive medications, was achieved. CLVs developed in more than thirty-nine percent of cases (15 eyes out of 38). In the present study, these cases were followed up further, and new cases were added if they fulfilled the inclusion criteria. The development of CLVs in this case study was observed in 50% of cases (29/58). The CLVs had characteristic lymphatic vessel appearances—they had uneven calibers, a sausage-shaped pattern, appeared in the form of plexus or solitary vessels running parallel to the limbus, perpendicular to it, or in a free manner, and had VLSs in their lumens in OCT scans. CLV identification by OCT in more than 82% of cases demonstrated that AH flowed from the SCS across the sclera to CLVs. Based on the clinical observations, a relationship between the duration of biomicroscopic CLV appearance and a decrease in IOP levels was established. It was concluded that longer CLVs identified biomicroscopically produced a poor prognosis for IOP control. Pronounced CLVs for a longer period indicated the possible resistance to fluid flow in the path ahead. The biomicroscopic appearance of CLVs may be an indication for initiating some kind of medical treatment to enhance conjunctival lymphatic outflow to control IOP levels and preserve visual functions.

Ocular lymphangiogenesis may explain some of the observations made in this study for the sites of CLV development, depending on the eye operated on. As presented in Table 7, CLVs developed predominantly in the nasal quadrants in the right eyes and in temporal quadrants in the left eyes. Ocular lymphangiogenesis determined that limbal and conjunctival lymphatic networks are progressively formed from a primary lymphatic vessel that grows from the nasal-side medial canthus region at birth. This primary lymphatic vessel immediately branches out, invades the limbus and conjunctiva, and bidirectionally encircles the cornea. As a result, the distribution of ocular lymphatics is significantly polarized toward the nasal side, and limbal lymphatics are directly connected to conjunctival lymphatics. New lymphatic sprouts are produced mainly from nasal-side limbal lymphatics, presenting the nasal side of the eye as more responsive to fluid drainage and inflammatory stimuli [66]. Further research is needed to explore the practical implications of the observations made in the present study.

The clinical observations and results of this study permit us to conclude that CLVs play an important role in decreasing IOP following surgery. The patency of CLVs is paramount to attain the success of any glaucoma surgery. Any resistance to lymphatic flow results in the failure of the procedure. Hence, it is wise to save the lymphatics not only at the time of surgery, but also before surgery. The instillation of hypotensive medications with preservatives has an adverse effect on the ocular surface and the status of conjunctival lymphatics [39,67,68]. The earlier the surgery is attempted, the higher the chances of lymphatics being functional, and the higher the chances of long-term surgical success rates.

The analysis showed that the main reason for failure in this study was the blockage of the trabeculotomy’s opening by iris tissue, leading to the elevation of IOP levels. The blockage of the trabeculotomy’s opening by iris tissue is a well-known complication of Nd:YAG laser goniopuncture [69]. To overcome this complication, we further modified the technique by performing a high-frequency peripheral iridotomy at the time of surgery.

*The mechanism of IOP decrease* may be explained as follows: AH bypasses resistance at the TM and the ciliary muscle and travels from the AC to the ISR through the trabeculotomy opening or percolates through the thinned inner wall of the SC. The healed-up conjunctiva prevents any flow to the subconjunctival space. In the ISR, AH accumulates temporarily and, after creating pressure above the episcleral vein pressure, it encounters the interstitial spaces of the uvea, where it acts as a trigger for the development of newly incorporated veins and lymphatic vessels. Surplus AH overfills the interstitial spaces and lymphatics. From the ISR, the AH enters the SCS to be absorbed by the vortex vessels or flows across the sclera via scleral pores or through intrascleral channels to be collected by orbital vessels or to drain into CLVs. When a balance is achieved between AH inflow from the AC to the ISR and SCS and AH outflow from the SCS, the CLVs decrease in size and number, leaving a few to maintain the fluid flow.

The proposed technique presents certain *advantages*. All surgical steps were performed without perforating the eyeball; hence, all of the complications related to the sudden decrease in IOP were either minimized or completely avoided. The decrease in IOP occurred without the formation of a bleb; therefore, complications related to blebs were negligible and completely avoided. As this technique was bleb-independent, the use of antimetabolites became irrelevant, and all adverse effects related to their use were avoided. The conjunctiva was intentionally permitted enough time to heal, preventing any flow to the subconjunctival space. The enhancement of AH outflow from the SCS to CLVs occurred via the natural outflow pathway.

Some of the *drawbacks* of this technique include its ab externo approach, the need for extensive tissue dissection resulting in increased surgical trauma, the prolongation of surgery time, two-stage surgery, and the need for the close monitoring of patients in the postoperative period. Blockage of the trabeculotomy by iris tissue may cause IOP elevation. A pre- peri-, or postoperative peripheral iridotomy helped to avoid this complication.

The study has certain *limitations*. It was a non-randomized, non-control interventional case series. It was not a clinical trial study. Randomized, controlled, and comparative studies with longer follow-up visits and larger groups are required to further confirm the efficacy and safety of this technique and to verify the proposed hypothesis of the IOP decrease mechanism.

## 5. Conclusions

The results of this interventional case series enabled us to conclude that the proposed technique is safe to perform and effective in decreasing IOP and reducing the use of hypotensive medication in glaucoma patients. IOP decreases without the formation of a filtering bleb. The technique enhances AH flow from the SCS to the circulatory system and extraocular lymphatics, and some of the AH flows from the SCS to CLVs through scleral pores or intrascleral channels. For predictable hypotensive outcomes, every precaution should be taken in glaucoma surgery to preserve lymphatics.

## 6. Patents

Patent of the Russian Federation for invention No. 2766730, dated 15 March 2022. Patent of the Russian Federation for invention No. 2782126, dated 21 October 2022.

## Figures and Tables

**Table 1:** Demographic and clinical data of 58 patients who underwent the study procedure.

Variables	Number of Patients (%)
Eyes n	
Right	28 (48.3)
Left	30 (51.7)
Sex	
Male	18 (31.1)
Female	40 (68.9)
Mean age (SD) [95% CI] years	75.5 (7.7) [73.5–77.5]
The median postoperative observation period (p^25^; p^75^) in weeks	76.3 (63.1; 111.8)
Type of glaucoma	
Primary open angle	42 (72.4)
Previously operated open-angle glaucoma	16 (27.6)
Glaucoma surgery performed one time	11
Glaucoma surgery performed two times	4
Glaucoma surgery performed more than three times	1
Type of glaucoma surgery performed (number of eyes)	
Trabeculectomy	6
Segmental dilation of Schlemm’s canal using stainless steel expander	7
Laser iridotomy	2
Cyclodialysis ab externo with implantation of a collagen implant in the tunnel	6
Non-penetrating deep sclerectomy	1
The time lapse between the previous glaucoma surgery and the study procedure	
<12 months	6
13–24 months	2
25–36 months	2
37–48 months	0
49–60 months	5
>60 months	1
Severity of glaucoma	
Moderate	21 (36.2)
Advanced	37 (63.8)
Median preoperative IOP in mm Hg (p^25^; p^75^)	27.0 (23.5; 28.0)
Median preoperative hypotensive medication use (p^25^; p^75^)	3 (2; 3)
Number of patients who used 2 classes of hypotensive medications (BAB + AAA = 2 Cases; BAB + CAI = 4 cases; BAB + PA = 9 cases; AAA + PA = 1 case; PA + CAI = 5 cases)	21 (36.2)
Number of patients who used 3 classes of hypotensive medications (AAA + BAB + CAI = 4 cases; BAB + CA + CAI = 1 case; AAA + BAB + PA = 2 cases; BAB + CAI + PA = 19 cases)	26 (44.8)
Number of patients who used 4 classes of hypotensive medications (AAA + BAB + CAI + PA)	10 (17.2)
Number of patients who used 5 classes of hypotensive medications (AAA + BAB + CA + CAI + PA)	1 (1.7)
Lens condition	
Visually significant cataract	40 (68.9)
Pseudophakia	15 (25.9)
Comorbidities	
Macular degeneration	3 (5.2)
Pseudo-exfoliative syndrome	24 (41.4)
High myopia	3 (5.2)
Diabetes mellitus	5 (8.6)

AAA = alpha-adrenergic agonists; BAB = beta-adrenergic blockers; CA = cholinergic agents; CAI = carbonic anhydrase inhibitors; CI = confidence interval; IOP = intraocular pressure; n = number of cases; p^25^ = 25th percentile; p^75^ = 75th percentile; PA = prostaglandin analogs; SD = standard deviation.

**Table 2:** IOP changes.

Follow-Up Period	n	IOP in mmHg Median (p^25^; p^75^); Mean (SD)	% Reduction in IOP	*p*-Value
Baseline	58	27.0 (21.0; 30)	-	-
1 day	58	14.7 (7.1)	45.6	<0.0001
1 week	56	9.0 (7.2; 13.0)	66.7	<0.001
1 month	57	11.3 (4.3)	58.1	<0.0001
3 months	49	12.5 (10; 16.0)	53.7	<0.001
6 months	51	14.9 (5.0)	44.8	<0.0001
12 months	43	15.0 (13.0; 18.0)	44.4	<0.001
18 months	30	16.5 (6.2)	38.9	<0.0001
24 months	16	15.2 (3.3)	43.7	<0.0001

IOP = intraocular pressure; n = number of cases; p^25^ = 25th percentile; p^75^ = 75th percentile; and SD = standard deviation.

**Table 3:** Patient distribution as a percentage reduction in IOP at different follow-up visits.

Reduction in IOP %	Follow-Up Visits, n (%)						
	1 m	3 m	6 m	9 m	12 m	18 m	24 m
≤20	1 (1.7)	5 (10.2)	8 (15.7)	5 (11.4)	7 (16.3)	5 (16.7)	1 (6.3)
>20 and ≤30	2 (3.5)	6 (12.2)	5 (9.8)	7 (15.9)	4 (9.3)	5 (16.7)	3 (18.7)
>30 and ≤40	6 (10.5)	4 (8.2)	8 (15.7)	6 (13.6)	10 (23.3)	9 (30.0)	5 (31.3)
>40	48 (84.3)	34 (69.4)	30 (58.8)	26 (59.1)	22 (51.1)	11 (36.6)	7 (43.7)
n	57	49	51	44	43	30	16

Where IOP = intraocular pressure; n = number of patients; m = months.

**Table 4:** Period between surgery and Nd:YAG laser trabeculotomy.

Period	Number of Cases
Laser trabeculotomy not performed	19
≤10 days	20
11–30 days	13
1–3 months	4
3–6 months	2
Total	58

**Table 5:** IOP decreases after Nd:YAG laser trabeculotomy procedure.

IOP (mmHg)	Number of Cases before Trabeculotomy	Number of Cases after Trabeculotomy
≤10.0	0	17
10.1–15	0	13
15.1–20	7	5
20.1–25	6	4
25.1–30	19	0
30.1–35	4	0
35.1–40	1	0
≥40	2	0
Total	39	39

**Table 6:** Time of CLV development and duration of CLV visibility based on slit-lamp examination in twenty-nine eyes.

CLVs Details	Follow-Up Visits, Number of Eyes								
	1 D	1 W	1 M	3 M	6 M	9 M	12 M	18 M	24 M
Time of CLV development	6	7	14	1	0	1	0	0	0
CLVs remained identifiable	3	2	11	5	1	2	1	0	4

D = day; W = week; M = months; and CLVs = conjunctival lymphatic vessels.

**Table 7:** CLV development as per the operated eye.

CLVs Observed at	Surgery Performed on the Right Eye, Number of Eyes		Surgery Performed on the Left Eye, Number of Eyes	
	Slit-Lamp Exam.	OCT Exam.	Slit-Lamp Exam.	OCT Exam.
Surgical site	1	2	1	1
Superior nasal quadrant	9	4	1	1
Superior temporal quadrant	0	1	4	0
Surgical site and superior nasal quadrant	2	6	1	5
Surgical site and superior temporal quadrant	0	1	2	2
Superior nasal and temporal quadrants	3	1	2	1
Surgical site and superior nasal and temporal quadrants	2	9	1	14
Total	17	24	12	24

CLVs = conjunctival lymphatic vessels; OCT = optical coherence tomography.

**Table 8:** CLV development and success rate.

Success Rate	Subgroup I * (n = 29), Number of Cases (%)	Subgroup II ** (n = 29), Number of Cases (%)	*p*-Value
Complete success	11 (37.9)	22 (75.9)	<0.001
Qualified success	24 (82.7)	26 (89.7)	0.59
Partial success	13 (44.8)	4 (13.8)	<0.0001
Failure	5 (17.3)	3 (10.3)	0.18

* Cases with CLV development; ** cases without CLV development.

## Data Availability

The data presented in this study are available upon request from the corresponding author. The data are not publicly available due to case histories of patients.

## Supplementary Materials

The following supporting information can be downloaded at: https://www.mdpi.com/article/10.3390/vision7030059/s1, Video S1_Case 2_ 1 month after surgery, CLVs in superior temporal quadrant. Video S2_Case 2_12 months after surgery, CLVs in superior nasal quadrant. Supplementary Table S1. Demographics, clinical features, pre- and postoperative observations of clinical cases having undergone study procedure as glaucoma component in combined surgery for coexisting pathology.
