# Supplementary material for: Clinical and Optical Coherence Tomography Evidence of Aqueous Humor Flow from the Suprachoroidal Space to Conjunctival Lymphatics
Source: Vision (Basel). 2023 Sep 5;7(3):59. doi: 10.3390/vision7030059 (PMC10536777; doi:10.3390/vision7030059)
Supplement: Supplementary file 1 [file vision-07-00059-s001.zip › Supplemental Table S1.pdf]

Supplemental Table S1. Demographics, clinical features, pre– and postoperative observations of clinical cases having undergone study procedure as glaucoma component in combined surgery for coexisting pathology.

Case	Age	Sex	Eye	Diagnosis	Baseline and postoperative observations														
						BCVA (logMAR)	IOP (mm Hg)	GM	Laser trabeculotomy	Slit-lamp examination				OCT examination				Follow-up (m)	Outcome
											CLVs				CLVs				
										Bleb	SNQ	STQ	SS	Bleb	SNQ	STQ	SS		
1	78	F	Rt	AG, IMC	Baseline	0.1	27	4										32	
					Postoperative				Yes, 1 time (21st day)										
					1 w	0	13	0		-	+	-	-	-	NP	NP	NP		Success
					1 m	0	13	0		-	+	+	-	-	+	+	-		Success
					3 m	0	9	0		-	+	+	-	-	+	+	-		Success
					6 m	0.1	11	0		-	+	-	-	-	+	-	-		Success
					12 m	0.05	11	0		-	+	-	-	-	+	+	+		Success
					18 m	0	15	0		-	+	-	-	-	+	+	-		Success
					24 m	0	15	0		-	+	-	-	-	+	-	-		Success
			Lt	AG, IMC	Baseline	0.1	27	3											
					Postoperative				Yes, 1 time (13th day)									27	
					1 w	0.15	6	0		-	-	-	-	-	-	+	-		Success
					1 m	0.15	10	0		-	-	-	-	-	-	+	+		Success
					3 m	0.15	10	0		-	-	-	-	-	-	-	-		Success
					6 m	0.2	15	0		-	-	-	-	-	-	+	-		Success
					12 m	0	15	0		-	-	-	-	-	-	-	-		Success
					18 m	0	10	0		-	-	-	-	-	-	-	-		Success
					24 m	0	10	0		-	-	-	-	-	-	-	-		Success
2	80	M	Lt	AG, IMC	Baseline	0.4	31	2										12	
					Postoperative				No										Success
					1 w	0	10	1		-	-	-	-	-	**NP**	**NP**	**NP**		Success
					1 m	0	14	0		-	-	+	-	-	+	+	+		Success
					3 m	0	10	0		-	-	+	-	-	**NP**	**NP**	**NP**		Success
					6 m	0	10	0		-	-	+	-	-	**NP**	**NP**	**NP**		Success
					12 m	0	10	0		-	+	-	-	-	+	-	+		Success

3	69	F	Lt	AG, HC, ERM	Baseline	1	30	3										15	
					Postoperative				Yes (4 times) 12th day, 19th day, 24th day and 80th day										
					1 w	0.2	24	2		-	-	+	+	-	-	+	+		Failure
					1 m	0.05	10	0		-	-	+	-	-	-	+	+		Success
					3 m	0.05	23.5	0		-	-	-	-	-	-	-	-		Failure
					6 m	0.05	34	3		-	-	-	-	-	-	-	-		Failure
					12 m	0.15	24	3		-	-	-	-	-	+	+	+		Failure

BCVA indicates best-corrected visual acuity; IMC, immature cataract, HM, hyper mature cataract; ERM, epiretinal membrane; AG, advanced stage glaucoma; IOP, intraocular pressure; GM, glaucoma medications (number of classes); CLVs, conjunctival lymphatic vessels; OCT, optical coherence tomography; SNQ, superior nasal quadrant; STQ, superior temporal quadrant; SS, surgery site; m, months; w, week; NP, examination not performed.
